# Identification of *Streptococcus suis* Meningitis through Population-Based Surveillance, Togo, 2010–2014

**DOI:** 10.3201/eid2207.151511

**Published:** 2016-07

**Authors:** Haoua Tall, Berthe-Marie Njanpop-Lafourcade, Didier Mounkoro, Loukoumane Tidjani, Kodjo Agbenoko, Issifou Alassani, Moussa Amidou, Stanislas Tamekloe, Kenneth G. Laing, Adam A. Witney, Jason Hinds, Mark P.G. van der Linden, Bradford D. Gessner, Jennifer C. Moïsi

**Affiliations:** Agence de Médecine Préventive, Ouagadougou, Burkina Faso (H. Tall);; Agence de Médecine Préventive, Paris, France (B.-M. Njanpop-Lafourcade, B.D. Gessner, J.C. Moïsi);; Agence de Médecine Préventive, Dapaong, Togo (D. Mounkoro, L. Tidjani);; Ministry of Health, Togo (K. Agbenoko, I. Alassani, M. Amidou, S. Tamekloe);; St. George’s, University of London, London, UK (K.G. Laing, A.A. Witney, J. Hinds);; RWTH Aachen University, Aachen, Germany (M.P.G. van der Linden)

**Keywords:** meningitis, zoonoses, Streptococcus suis, occupational health, Africa, Togo, bacteria, surveillance, meningitis belt

## Abstract

During 2010–2014, we enrolled 511 patients with suspected bacterial meningitis into surveillance in 2 districts of northern Togo. We identified 15 persons with *Streptococcus suis* infection; 10 had occupational contact with pigs, and 12 suffered neurologic sequelae. *S. suis* testing should be considered in rural areas of the African meningitis belt.

*Streptococcus suis*, an encapsulated, gram-positive bacterium, commonly colonizes the respiratory, genital, and intestinal tracts of pigs and may cause severe disease, including meningitis, sepsis, and bronchopneumonia (*1*). Zoonotic cases of *S. suis* invasive disease have been documented in Europe, Asia, the Americas, and Oceania among persons in direct contact with pigs or pork meat; the case-fatality rate is 10%–20%, and neurologic sequelae frequently occur (*2*–*4*). Two large outbreaks have occurred in China (*5*,*6*), but little is known about the disease among humans in Africa.

We began surveillance in May 2010 for acute bacterial meningitis in hospitals in 2 rural districts in Togo. The National Ethical Committee of Togo reviewed and approved our study protocols.

## The Study

During 2010–2014, we conducted surveillance for patients with signs and symptoms of meningitis at 5 hospitals in northern Togo: 4 in Dapaong, Tône District, and 1 in Cinkassé, Cinkassé District. These districts are within the African meningitis belt and experience annual outbreaks of hyperendemic bacterial meningitis during the dry season (November–April) and generalized epidemics every 4–6 years (*7*). Lumbar puncture was performed at admission on all patients with suspected meningitis, provided informed consent had been given. Cerebrospinal fluid (CSF) specimens were transferred to the regional bacteriology laboratory in Dapaong for cytologic testing, Gram staining, latex agglutination, and culture. CSF samples were further tested by conventional PCR at Centre Muraz Laboratory (Bobo-Dioulasso, Burkina Faso) or Institut National d’Hygiène (Lomé, Togo) for identification of *S. pneumoniae*, *Neisseria meningitidis*, and *Haemophilus influenzae* type b (Hib).

Beginning in August 2011, *Streptococcus* isolates from Dapaong were stored at −80°C in STGG (skim milk, tryptone, glucose, glycerol) medium and sent to the National Reference Center for Streptococci (Aachen, Germany) for confirmatory testing. In April 2013, after the reference laboratory identified several cases of *S. suis* infection, the bacteriology laboratory in Dapaong implemented additional diagnostic testing using the API Kit (bioMérieux, Marcy l’Etoile, France) to enable rapid case detection by culture. For species confirmation and molecular typing, we sequenced the genome of *S. suis* isolates by using the Nextera XT DNA Library Preparation Kit (Illumina, San Diego, CA, USA) and the MiSeq Reagent Kit v3 (Illumina) for 2 × 300-bp paired-end reads. We deposited sequence data in the European Nucleotide Archive (accession no. PRJEB12952).

In June 2014, we visited all identified *S. suis* meningitis case-patients and used a questionnaire to collect data on their environments and contact with pigs and pork meat. Thereafter, we prospectively administered the questionnaire to new case-patients. We used Stata 12 (StataCorp LP, College Station, TX, USA) to analyze the data. 

During August 2010–July 2014, we enrolled 511 persons with suspected bacterial meningitis, of whom 126 (24.6%) were <5 years of age. We performed lumbar puncture on 489 enrollees: 89 were positive for *S. pneumoniae*, 60 for *N. meningitidis*, 15 for *S. suis*, 9 for *Streptococcus* sp., 8 for Hib, and 7 for other identified pathogens; 301 had no identified etiology. *S. suis*–positive cases were confirmed by genome sequence analysis of the isolates, using Kraken (*8*) and comparative analyses with reference *S. suis* genomes (*9*) and other outlier species. We predicted that all 15 *S. suis* isolates were serotype 2 due to 100% coverage of the associated capsular polysaccharide loci sequence (*10*); 6 were sequence type 1, and 11 were a single-locus variant of sequence type 1 exhibiting a new *rec*A allele sequence (http://ssuis.mlst.net/).

*S. suis* cases peaked in April through August each year (Figure). Of the 15 *S. suis* patients, 3 were 5–14 years of age, 7 were 30–49 years of age, and 5 were >50 years of age; 12 (80%) patients were male (Table 1). Median time from symptom onset to hospitalization was 2 days (interquartile range 1–4 days), similar to the time for patients with meningitis caused by other pathogens. One (6.7%) patient died; 12 (85%) of the 14 survivors had neurologic sequelae (Table 1). Resistance to antimicrobial drugs was relatively uncommon (Table 2).

**Figure:**
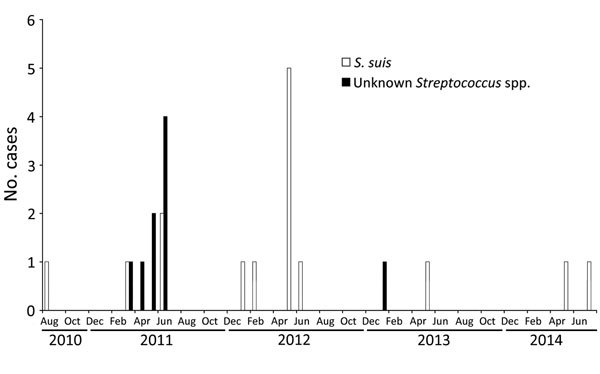
*Streptococcus suis* and other *Streptococcus* spp. infections identified per month through acute bacterial meningitis surveillance in northern Togo, 2010–2014.

**Table 1:** Characteristics of patients from 2 rural districts with meningitis caused by various pathogens, Togo, 2010–2014

Characteristic	No. (%) case-patients infected with					No. (%) all case-patients, N = 489
	*S. suis*, n = 15	Other *Streptococcus* sp., n = 9	*S. pneumoniae*, n = 89	Other infections, n = 75*	No etiologic agent, n = 301	
Age, y						
<5	0	3 (33)	12 (13)	22 (29)	84 (28)	121 (25)
5–14	3 (20)	0	34 (38)	36 (48)	74 (25)	147 (30)
15–29	0	2 (22)	23 (26)	7 (9)	65 (22)	97 (20)
30–49	7 (47)	4 (44)	13 (15)	8 (11)	52 (17)	84 (17)
>50	5 (33)	0	7 (8)	2 (3)	26 (9)	40 (8)
Sex						
M	12 (80)	6 (67)	42 (47)	39 (52)	156 (52)	255 (52)
F	0 (0)	3 (33)	47 (53)	36 (48)	145 (48)	244 (48)
Died	1 (7)	1 (11)	29 (33)	5 (7)	41 (14)	77 (16)
Sequelae						
Any†	12 (80)	2 (22)	17 (19)	5 (7)	27 (9)	63 (13)
Hearing loss‡	8 (67)	1 (50)	8 (47)	3 (60)	4 (15)	24 (5)
Paralysis‡	2 (17)	1 (50)	3 (18)	2 (40)	6 (22)	14 (3)
Visual impairment‡	5 (42)	0	2 (12)	0	1 (4)	8 (2)
Seizure disorder‡	0	0	0	0	2 (7)	2 (0)

*Includes 60 case-patients with *Neisseria meningitidis*, 11 with *Haemophilus influenza*e, 1 with *Escherichia coli*, 1 with *Staphylococcus aureus*, 1 with *Streptococcus pyogenes*, and 1 undetermined. †Percentages calculated among all patients. ‡Percentages calculated among patients with sequelae.

**Table 2:** Antimicrobial resistance patterns of *Streptococcus suis* and other *Streptococcus* spp. isolates from meningitis patients in 2 rural districts in northern Togo, 2010–2014

Antimicrobial drug	No. *S. suis* samples tested/no. (%) susceptible		No. other *Streptococcus* spp. samples tested/no. (%) susceptible
	Tested at local laboratory*	Tested at reference laboratory†	
Amoxicillin	15/15 (100)	11/10 (91)	9/5 (56)
Cefotaxime	0	11/10 (91)	0
Ceftriaxone	15/15 (100)	0	9/8 (89)
Chloramphenicol	15/12 (80)	11/11 (100)	9/8 (89)
Clindamycin	0	11/10 (91)	0
Cotrimoxazole	15/14 (93)	0	9/6 (67)
Gentamicin	15/7 (47)	0	9/4 (44)
Levofloxacin	0	11/10 (91)	0
Oxacillin	12/3 (25)	11/11 (100)	1/0
Penicillin	15/15 (100)	11/10 (91)	9/5 (56)
Rifampin	15/15 (100)	0	9/6 (67)
Tetracycline	0	11/0	0

*The regional bacteriology laboratory in Dapaong, Togo. †National Reference Center for Streptococci in Aachen, Germany.

All 15 *S. suis* meningitis patients were involved in pig farming or slaughtering or had a family member who was: 3 each were pig farmers only or butchers only, 3 were pig farmers and involved in slaughtering, and 6 had a family member engaged in 1 of these activities. Twelve patients reported handling pig meat during cooking; 14 reported eating pork (9 at least once per week). Three patients reported that a family member or neighbor had also contracted meningitis and had subsequent hearing loss.

## Conclusions

In the area of Togo under surveillance, meningitis cases peak during the dry season, and *S. pneumoniae* and *N. meningitidis* have been the leading causal agents since the introduction of Hib conjugate vaccine in 2008. During 2010–2014, we identified 15 cases of *S. suis* meningitis, representing 3.1% of all suspected bacterial meningitis cases and 8.0% of etiologically confirmed cases. These numbers are probably an underestimate because to identify *S. suis*, we relied on culture only, whereas we used CSF PCR to test for other bacteria. Furthermore, 9 *Streptococcus* isolates did not survive to be sent for confirmatory testing and may represent additional *S. suis* cases.

Our investigation showed that two thirds of *S. suis* meningitis patients were involved in pig farming or slaughtering, and the remainder had a family member who was involved in these activities. Most case-patients regularly cooked and ate pork. *S. suis* meningitis cases appear to have a seasonal pattern, clustering in May–July, a period of intensive slaughtering, during which pigs are confined to pens, and run-off water accumulates nearby in open-air pits. Slaughtering is conducted in butcher shops, where carcasses remain for several days. Approximately 30 pork butchers work in Dapaong, and overall, >1,000 pigs are slaughtered in Tône District each year (G. A. Boukaya, Direction Régionale de l’Agriculture, de l’Elevage et de la Pêche des Savanes, pers. comm., 2015 Aug 1). Based on these data, the cumulative incidence of *S. suis* meningitis among Dapaong butchers during 2010–2014 was 20% (6 cases/30 butchers), compared with 0.00375% (15 cases/400,000 total population) in Tône and Cinkassé Districts (incidence rate ratio 5,333). Although this was not a rigorously controlled prospective study, our results are highly suggestive of an association between butchering pigs and acquisition of *S. suis* meningitis; pig contact through farming or cooking may also be a risk factor. Three patients reported that a close contact had also contracted meningitis; these cases probably reflect acquisition from a shared environmental source.

We plan to continue laboratory testing for *S. suis* among patients enrolled in bacterial meningitis surveillance in northern Togo and investigating risk factors among confirmed case-patients. Biochemical testing for speciation of *Streptococcus* spp. is rarely part of routine bacteriologic evaluation of CSF in Africa and was not done at our site before the initial identification of several *S. suis* cases. *S. suis* testing should be considered for meningitis patients in areas of rural Africa where pig farming is common. All *S. suis* isolates in our study were susceptible to ceftriaxone, the presumptive therapy for nonepidemic meningitis in the meningitis belt; consequently, our findings do not suggest a need for altering current therapeutic guidelines. To reduce the incidence of *S. suis* meningitis, future efforts should first more fully delineate the practices that increase the risk for infection and then be directed toward educational campaigns targeting groups at high risk.

## References

[1] Gottschalk M. Streptococcosis. In: Straw BE, Zimmerman JJ, D’Allaire S, Taylor DJ, editors. Diseases of swine. 10th ed. Ames (IA): Blackwell Publishing; 2012. p. 841–55.

[2] Goyette-Desjardins G, Auger JP, Xu J, Segura M, Gottschalk M. *Streptococcus suis*, an important pig pathogen and emerging zoonotic agent—an update on the worldwide distribution based on serotyping and sequence typing. Emerg Microbes Infect. 2014;3:e45. 10.1038/emi.2014.45 PMC4078792 26038745

[3] Segura M, Zheng H, de Greeff A, Gao GF, Grenier D, Jiang Y, Latest developments on *Streptococcus suis*: an emerging zoonotic pathogen: part 1. Future Microbiol. 2014;9:441–4 . 10.2217/fmb.14.14 24810343

[4] Segura M, Zheng H, de Greeff A, Gao GF, Grenier D, Jiang Y, Latest developments on *Streptococcus suis*: an emerging zoonotic pathogen: part 2. Future Microbiol. 2014;9:587–91 . 10.2217/fmb.14.15 24957086

[5] World Health Organization. Outbreak associated with *Streptococcus suis* in pigs, China. Wkly Epidemiol Rec. 2005;80:269–70. 16116899

[6] Yu H, Jing H, Chen Z, Zheng H, Zhu X, Wang H, Human *Streptococcus suis* outbreak, Sichuan, China. Emerg Infect Dis. 2006;12:914–20 . 10.3201/eid1206.051194 16707046 PMC3373052

[7] Greenwood B. Editorial: 100 years of epidemic meningitis in West Africa—has anything changed? Trop Med Int Health. 2006;11:773–80 . 10.1111/j.1365-3156.2006.01639.x 16771997

[8] Wood DE, Salzberg SL. Kraken: ultrafast metagenomic sequence classification using exact alignments. Genome Biol. 2014;15:R46 . 10.1186/gb-2014-15-3-r46 24580807 PMC4053813

[9] Holden MT, Hauser H, Sanders M, Ngo TH, Cherevach I, Cronin A, Rapid evolution of virulence and drug resistance in the emerging zoonotic pathogen *Streptococcus suis.* PLoS One. 2009;4:e6072 . 10.1371/journal.pone.0006072 19603075 PMC2705793

[10] Okura M, Takamatsu D, Maruyama F, Nozawa T, Nakagawa I, Osaki M, Genetic analysis of capsular polysaccharide synthesis gene clusters from all serotypes of *Streptococcus suis*: potential mechanisms for generation of capsular variation. Appl Environ Microbiol. 2013;79:2796–806 . 10.1128/AEM.03742-12 23416996 PMC3623174

